# Compliance and initiative: A discussion on the relationship between standards and activities in radiopharmaceutical production

**DOI:** 10.1016/j.heliyon.2024.e26003

**Published:** 2024-02-12

**Authors:** Gerferson André Silva Costa, Julie Micheline Amaral Silva, Raquel Guimarães Soares, Celina Maria Modena, Fabiana Goulart de Oliveira, Francisco de Paula Antunes Lima

**Affiliations:** a Instituto René Rachou, Fundação Oswaldo Cruz, Fiocruz Minas, Belo Horizonte, Brazil, Av. Augusto de Lima, 1715, Barro Preto, Belo Horizonte, Minas Gerais, 30190-002, Brazil; b Universidade Federal de Minas Gerais, Av. Antônio Carlos, 6627, Campus Pampulha, Zip Code, 31.270-901, Belo Horizonte, Minas Gerais, Brazil; c Universidade do Estado de Minas Gerais, Divinópolis, Brazil. Av. Paraná, 3001, Jardim Belvedere, Divinópolis, Minas Gerais, Brazil

**Keywords:** Radiopharmaceuticals, Activity analysis, Adherence to standards, Risk control

## Abstract

This study investigated how a standard could become reality-based in a workplace where certain types of deviations are not permitted, such as a radiopharmaceutical production unit. Compliance with standards is necessary to ensure the safety of individuals who manufacture such substances as well as the security of patients receiving treatment. In this qualitative case study, an ergonomic analysis of work (also known as activity analysis) was performed, with noncompliance recorded in internal audits as a starting point: the lack of double-checking in radiopharmaceutical synthesis cassette assembly. Field observations and self-confrontation interviews with workers from a production unit were conducted to analyze the activities. Although a double-check did not occur, the radiopharmaceutical synthesis operator apparently developed another risk control strategy, focusing his attention to the equipment assembly details, which necessitated continuous control and verification actions to ensure that there were no problems at this stage of production. A multilevel approach was used to demonstrate how the safety and quality of production processes based on standard compliance only become effective and adherent to the activity after resolving various conflicts at work, including control systems (external and internal audits), work collectives, the contradiction of the activity itself, and the discussion of singular situations arising daily. This study contributes to the discussion on workplace safety considering standardization and advances the discussion on changing perspectives regarding rule compliance.

## 1 Introduction

One of the most noticeable characteristics of ultrasafe systems is prevention through mandated standards and processes. Although these tools are significant for increased safety, they are often criticized for the excessive rules [1,2]. This poses the challenge of reconciling mandatory standards with reduced procedures, allowing operator autonomy, initiative, and discretion. Some authors in the critical literature on safety proposed a revaluation of professionalism, combining autonomy, responsibility, and expertise, while reducing the weight of mandatory procedures [1]. Others proposed integrated safety, combining standardized safety with safety in action [3,4], with different weights assigned to the two poles depending on the nature of the system [5]. Compliance behavior is a desired outcome in ultrasafe systems [5]. These systems invest in supervising frontline workers. This supervision seeks to minimize workers' exposure to a few hazardous conditions. When accidents occur, the causes are investigated to reduce or even eliminate future risk exposure [6].

Undoubtedly, in addition to technical reliability, management systems have contributed to the improvement of work and production process safety. However, the existence of excessive rules and procedures is broadly recognized [1,4,[7], [8], [9]]. Various factors cause this phenomenon, including the presence of superfluous, outmoded, contradictory, or even counterproductive processes [2,4], some of which generate other hazards while preventing others. This has resulted in initiatives aimed at minimizing the number of obligatory procedures.^1^

Other authors [10] have suggested that the core point of a rules management system is knowing how to deal with rule diversity, exceptions [10,11], and rule expiration [10]. The more a collection of rules falls under the obligation of legal processes within the purview of audits, the less chance organizations have to retain the flexibility of their set of rules to deal with real-work diversity [10]. Regarding rules and their relationship with autonomy, some claim that the competencies of rule users must be considered; the more competent they are, the less reliant they are on action rules [12]. After all, “safety is created not by rules, but by what people actually do" [13]. However, under this generic skills-based approach, it is worth examining which specific abilities are necessary to balance compliance and autonomy to guarantee quality and safety.

This study focuses on radiopharmaceutical production, wherein stringent procedures are necessary to guarantee the health of the workers and that of the patients who receive the medications. This study revisits the issue of operator autonomy in ultrasafe systems subject to imperative norms and answers the following questions: How can one identify the areas of autonomy of operators who carry out work that goes beyond just following instructions and requires initiative, inventiveness, and improvisation in the face of unforeseen circumstances? What could be the discrepancy between prescribed work and real work [4] (or work-*as*-imagined vs. work-*as*-done in Sidney Dekker's terms [1]) in high-risk enterprises where particular norms are not bureaucratic but critical for technological reasons? Considering the rising demand for radiopharmaceuticals, this problem has social relevance and is significant for developing ultrasafe systems [14,15].

Radiopharmaceuticals are used in nuclear medicine for cancer diagnosis and treatment [[16], [17], [18], [19]]. Its manufacturing occurs in regulated settings with physicochemical procedures and labor that are standardized in terms of safety, radiological protection, and quality. Brazil currently has 13 production facilities, four of which are federal government laboratories, and nine are owned and operated by private businesses [20]. The Brazilian Nuclear Policy aims to protect national sovereignty by supporting the growth of the domestic radiopharmaceutical industry and establishing guidelines for planning, taking action, and engaging in nuclear and radioactive activities [21]. Investigations using radiopharmaceuticals for diagnosing and treating cancer and other diseases have sparked new research worldwide, demonstrating their efficacy and how well they align with regulatory bodies' goals of maintaining patient and worker safety [14,15,19,22].

Radiopharmaceutical manufacturing facilities undergo several audits to establish their position in the market and verify the requirements established by regulatory organizations and the ISO 9001 series [23]. To ensure that the work is completed as planned, this external and internal control system identifies deviations from norms, also known as nonconformities or anomalies, and corrects them to close the gap between work-*as*-imagined and work-*as*-done [1]. This system also tends to eliminate initiative behavior in favor of conformity. This control mechanism eventually selects recurring deviations that are not addressed by traditional techniques, such as retraining or punishment. This study examined a case that precisely represents a circumstance that raises this paradox: the failure to perform a double-checking procedure during the assembly of equipment for the final production of medicines, regarded as a noncompliance that endangers the patient's health and life. This inaccuracy is unacceptable in radiopharmaceutical production. Thus, the double-checking rule was adopted at the workers' recommendation despite the fact that, paradoxically, the employees themselves do not follow it.

The standardization of work processes accompanies industrial development, reaching a climax in Total Quality procedures and the diffusion of ISO standards. In addition to the notion that a production system can be completely managed using procedures, its exponential expansion results from an ad hoc logic that develops a new process for each deviation or unexpected event without considering its overall relevance and coherence. This is a management style for businesses seeking continuous improvement in organizational performance through frequent product and production reviews [24]. Historically, this management type has enabled the advancement of “best practices" for arranging production processes, providing services, and preventing recurring faults at work. However, excessive standards created by this production management method have a detrimental impact on occupational safety [2].

In contrast to excessive standardization, resilience engineering views errors as unavoidable and indicates that systems should focus on controlling variability rather than standardization. Managers must reinforce the variability that leads to expected results, while eliminating variability that leads to undesirable results. This final principle assumes that frontline staff are capable and autonomous decision-makers [25]. In addition to avoiding criticizing workers (which is frequently viewed as a source of risk in traditional approaches), this perspective understands that the diversity of daily performance requires constant modifications in task execution. This supports what has already been stated: rather than organizations responding creatively to “failures" in the manufacturing process, it is necessary to continuously review events and incidents, revisit and understand successful actions, and understand humans as resources required for system flexibility and resilience [7,26].

Following the proposal of Daniellou et al. [4] that initiative and compliance behavior do not contradict, and that compliance also results from workers' initiative,^2^ we argue, based on the analyzed case, that compliance, consensually considered to be necessary in high-risk systems, is obtained in practice when conflicts are resolved at different levels: (i) at the level of the internal and external control system, which is intolerant of deviations by definition; (ii) at the level of the work collective, which must hold an additional conference in addition to its regular operations; (iii) when the worker acknowledges that additional verification is essential but remains insufficient or unreliable within the context of the activity itself; and (iv) in the presence of particular circumstances that only employees can understand.

We argue that compliance with norms is possible only when these norms conform to individual and collective activities. From a theoretical perspective, we examine the reciprocal rapprochement between norm and activity as an integrated four-fold movement of development of the adherence [27] of norms to the activity, collective, quality assurance system, and specific work situation. This provides a better understanding of integrated safety and how to create it, whose upward and downward movements (autonomy, initiative, and creativity) derive coherence from what occurs at the activity level. These integration processes (among which, this study deals specifically with the “convergence problem”) remain relatively undetermined in the literature on integrated safety [3,4,6]. More than a “propensity” or a “motivation” to accept and apply the norms, it is the pertinence produced in the individual and collective activity that gives coherence to the initially conflicting requirements of the different levels. This study aims to understand how a standard can be made compliant with the activity (in the sense of being relevant and practicable) in an ultrasafe environment by discussing the problem of convergence and the inconsistencies highlighted by the literature on conformity and deviation.

The remainder of this paper is organized as follows: Section 2 discusses the methodological considerations that enabled the research on which this study is based; Section 3 presents the main empirical results — the production room and division of labor, assembling the cassette and caring for the fittings, the connection checking process and absence of double-checking, and the debate at existing conferences; Section 4 discusses the results and the theoretical –practical contribution to the worker health/ergonomics field; and Section 5 concludes and suggests future research directions.

## 2 Material and methods

### 2.1 Method of investigation

An ergonomic analysis of work activity (EAWA) [28] was conducted in a radiopharmaceutical production unit (RPU) that belonged to the autarchy of the federal government as part of this study using a qualitative approach [29,30]. An EAWA was conducted with the need for analysis of noncompliance and absence of double-checking by a second person in the assembly of radiopharmaceutical synthesis cassettes considered to accomplish the suggested objectives. Initial staff interviews, an examination of the institution's records (including the noncompliance report and the prescription for the synthesis of radiopharmaceuticals), and broad observations of the operation of the unit, from receiving client orders to dispensing the radiopharmaceuticals for transport comprised the exploratory stage. Subsequently, observations and interviews with the workers during the synthesis and fractionation of radiopharmaceutical doses were conducted to understand how the activities were carried out and how they interacted in this process. The succeeding stage focused on the synthesis of the radiopharmaceutical. This stage involved observations and self-confrontation interviews with the official synthesis operator to assess his performance during the cassette assembly and the conference process. Finally, diagnoses and recommendations were made in collaboration with the operators (officers and substitutes) in the final stage.

### 2.2 Ergonomic analysis of work activity

Ergonomic actions generally arise from demands presented by workers or company managers. Faced with the demand, the ergonomist must follow a few steps to analyze the work activity and propose relevant interventions: 1) analysis and reformulation of the demand based on the initial collection of information about the company operation, worker characteristics, and the place of work and other indicators; 2) analysis of the task, that is, the prescribed work; 3) open observations of the activity (how the work is done); 4) formulation of pre-diagnosis and definition of a systematized observation plan; 5) systematic observation of work activity, data processing, and validation; 6) local diagnosis with a description of the activities analyzed; and 7) formulation of recommendations [28,31].

In this study, the RPU quality assurance manager presented the demand. This was a nonconformity identified in the internal audit of the unit. The following sentence appears in the reported description of the “root cause" of noncompliance with reference to the lack of double-checking in the radiopharmaceutical production procedure:

“Since the process for building the fludeoxyglucose reagent kit is repetitive and carried out multiple times a week, the non-compliance is linked to a certain 'automatism' that eventually occurs in carrying out the production routine. Though trained and confident in their work, production technicians should continue to follow the checking procedure to prevent any failures.” (RPU Noncompliance report, 2020)

**Fig. 1:**
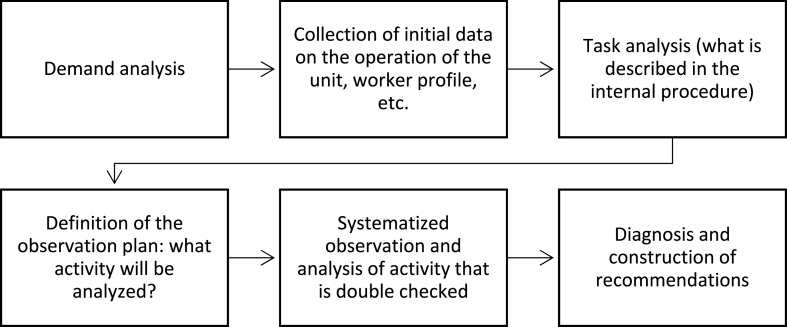
Ergonomic Work Analysis Flowchart at the RPU, 2020–2021 (Source: prepared by the authors).

Based on this noncompliance, the AET illustrated in the flowchart in Fig. 1 was performed and is described in detail in the following subsections.

### 2.3 Demand analysis

The quality assurance manager presented the socially expressed demand as a non-conformity. A problem had been identified in an internal audit that had yet to be resolved: the failure to perform an internal procedure during the radiopharmaceutical production phase—double-checking. Because a demand can only express a portion of the problem, providing it with additional information about the work environment, history of the demand, challenges that the demand faces, and specific representations that the workers involved in the production process have [28,31]. The methodology of this study is based on these preliminary findings and the subsequent expansion.

**Fig. 2:**
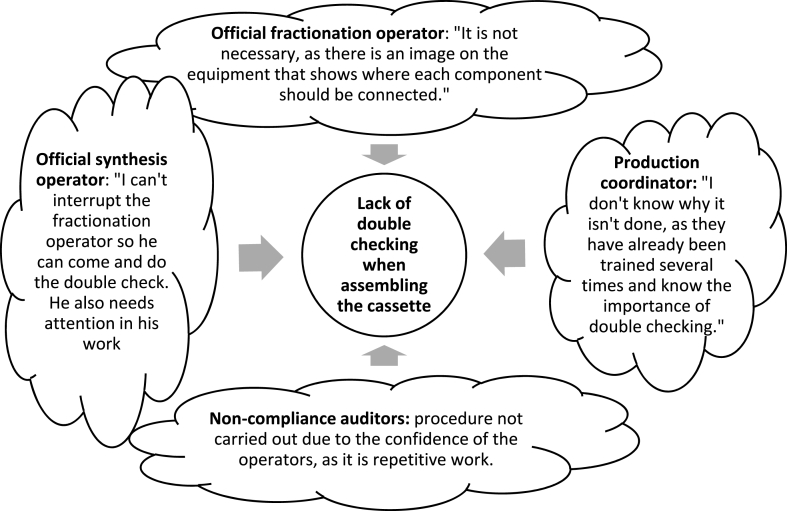
Different perspectives of RPU workers on the initial demand in 2020–2021. (Source: prepared by the authors).

The demand analysis included preliminary discussions with RPU workers to learn about the elements behind the presented demand that may aid in defining an object of analysis and formulating an action plan. Fig. 2 presents various perspectives on the demand.

Considering the highlighted elements, we made an initial reformulation of the demand, which would be investigated further with data from the research field: double-checking, despite being required by the RPU managers, is documented in an internal procedure, and the workers are properly trained; it does not appear to be practicable considering the time constraints—at the time of carrying out the double conference, the fractionation operator is busy carrying out his activities, and he does not believe in the usefulness of this action.

With this reformulation, the following questions were raised to guide the next steps of this study.

1. How is the equipment that synthesizes the radiopharmaceutical prepared?
2. How would a double conference contribute to this stage of production?
3. How is work divided in the production room?

In this way, we sought to understand how the RPU works as a whole and focused on the activities carried out in the workplace where the demand originates: the production room. The most in-depth activity in this analysis was the cassette assembly and the preparation of the equipment that synthesizes the radiopharmaceutical. Thus, this study answers the above questions and proposes sensible recommendations.

### 2.4 Data collection

To understand the demand, video conference interviews were held with the production coordinator and temporary unit manager. The first interview aimed to understand the function of the unit and determine the worker profiles. These interviews began with the question, “Can you describe how the production of radiopharmaceuticals is organized and who/how many workers are involved in each process?” Given the limitations of virtual interviews, further research was not conducted because the EAWA focused on real work. Field observations and interviews at the radiopharmaceutical production facility were made possible by improvements in Covid-19 monitoring indicators in the municipality.

During the initial observations and interviews, the activities of operators (synthesis and fractionation) were analyzed. After analyzing how double-checking was organized (or not) during the operator activities, the systematic interviews focused on radiopharmaceutical synthesis. This deepening was necessary to understand the need for an established internal checking procedure. Field observations enabled interviews during self-confrontation, which enhanced our understanding of the real work. This is based on a previous study which stated that “[…] without an analysis of the activity encompassing its bodily and tacit dimension, what remains is the analysis of the task, incapable of encompassing all the complexity of human activity” [32]. The interviews were guided by observations; therefore, they were unstructured. Based on the general question “What is happening here?“, which guided the open observations, we sought to understand the workers’ actions and their reasoning with the question “What are you doing right now?“.

To support the interviews, all observations during or after production were documented in a field diary. The worker could not be easily interrupted at all times to inquire about what he was doing because the cassette manufacturing process required focus, as will be discussed later. Images and films were used in addition to field diary entries to capture the workers’ gestures and postures while they carried out their activities and enable interviews in a series of self-confrontations.

### 2.5 Study participants

Because this study aimed to comprehend the cassette assembly process, the official radiopharmaceutical synthesis operator was the main subject of systematized observations and interviews. The production coordinator and other workers participated in the self-confrontation process to discuss how to put them back in position, debate how to assemble the cassette, and bring up the conference process when it happens. The interview in self-confrontation allows the implicit components of the activity to emerge that go beyond subjectivist explanations or common sense; it summarizes the perceptions, experiences, decisions, thoughts, feelings, and actions of the subjects in the activity [32]. In adapting the prescribed work to possible work, that is, in the work in action, the workers rescue the moment of their activity while envisioning themselves in a scenario, raising crucial issues. Consequently, the employee becomes his own work analyzer [33,35].

The production coordinator, substitute synthesis operators (two workers), official fractionation operator (who performs the cassette assembly check and has completed the synthesis process in exceptional circumstances), and official synthesis operator participated in the cross-self-confrontation interviews. The primary materials used in the cross-self-confrontation interviews with all participants were a table listing the radiopharmaceutical synthesis activities and photographs showing the activities performed, both of which were derived from field observations. The self-confrontation and videoconference interviews took 48 h to complete.

Considering that noncompliance is related to the failure to carry out an internal procedure in the production room, all operators were included in the self-confrontation interviews. We divided the operators into two phases based on the following reasoning.

• Phase 1: Conduct observations and interviews in self-confrontation with official production operators as they meet more than 80% of the annual production demand.
• Phase 2: Conduct interviews in self-confrontation with all production operators (officials, substitutes, and coordinators) using the activity analyzed in Phase 1 as a reference. The objective of this phase was to determine the different ways of developing the activity and debate the successful actions.

### 2.6 Data analysis

The data from the observations and interviews were stored in separate files (one for each worker), and traces of the observations (employees' words, gestures, actions, and incidents) were used to guide the interviews. The materials were repeated read to extract and analyze the meaning of the data collected, and themes were described to support the discussion of this research and answer the questions initially posed [29,30]. At the end of each file, themes that arose from the interview transcriptions or notes on the observations were explained. No software was used to analyze the interviews or field observation notes. This study was conducted in accordance with the accepted ethical norms..^3^

## 3 Results

### 3.1 Understanding the manufacturing area and workflow

Two personnel work in the radiopharmaceutical production room: a synthesis operator and a dose fractionation operator. The operator in charge of radiopharmaceutical synthesis assembles the cassette,^4^ which carries out the necessary physicochemical reactions. The assembly takes approximately 15–20 min. Majority of the cassette preparation are performed manually and involve the attachment of connectors, reagents, syringes, and filters. Following the cassette connection, the subsequent processes, which involve pressurizing and solubilizing the reagents, are automatic: moving the solution to the reactor where synthesis occurs, sending the finished radiopharmaceutical to the dose fractionator, and coupling the syringes to the couplers. If an error is found during automatic testing, the software alerts the user to a potential issue and/or locations that need to be examined (when dealing with loose cassette connections). The fractionation operator must verify that the cassette is correctly built before synthesis begins, that is, immediately following the cassette setup and connection to the module. There is no set protocol for this conference according to the RPU. Unit managers support employee training during the assembly phase. Based on prior experience as an operator (both synthesis and fractionation), the production coordinator typically leads the training.

The fractioning operator must prepare the equipment that divides and fills the doses for each client. This preparation includes filling the circuit with saline solution and inspecting for any potential leaks to the location where the radiopharmaceutical is packaged. The dose recipe for each client is entered into the equipment software, which is another crucial phase of the operation. The synthesis operator must verify that the filling is accurate after entering the doses in accordance with the production coordinator's calculations.

As previously stated, a lack of double-checking was discovered following the cassette assembly; thus, the primary activity under investigation was related to the radiopharmaceutical production procedure.

### 3.2 Cassette assembly activity: paying attention to how the connectors go properly

The first step in the cassette assembly is to remove it from its packing and tighten loosened connections. The operator claims that the sufficiency of his “grip" originates from his tactile perception:

*“I can feel the friction of the plastic material. I can't force it if it doesn't spin anymore; otherwise, I'll shatter the cassette."* (Chagas, radiopharmaceutical synthesis operator).

**Fig. 3:**
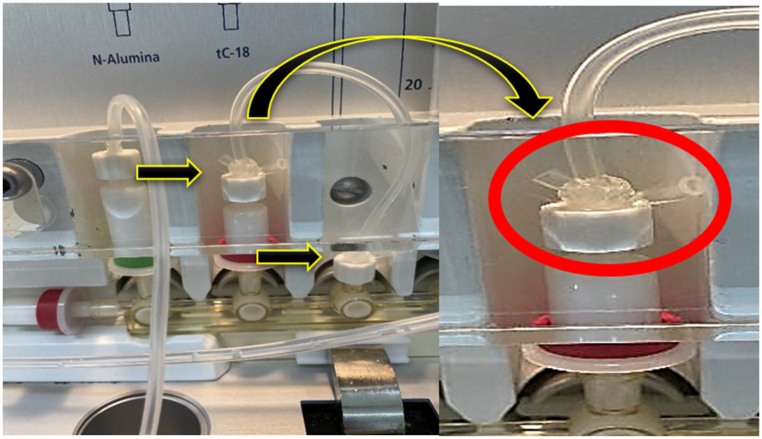
“Knots” tied in the fragile parts of the cassette, RPU, 2020–2021.

After tightening the connections, the operator ties the knot (tying a piece of the circuit–plastic material) to prevent the circuit from breaking during the synthesis, with appropriate pressurization for reagent circulation in the cassette. The knots are tied such that they did not block the flow of substances (Fig. 3).

“The knot must be on the rigid plastic joint, where it will not strangle the circuit. You tie a knot without tightening it and drag it down to rest on top of the plastic joint, then tighten the knot by pushing the ends together and cutting what's left with a box cutter. You must be careful not to cut the circuit when cutting, or I will have to replace the entire cassette." (Chagas, radiopharmaceutical synthesis operator)

Following these initial procedures and connecting the cassette to the equipment, the operator connects the radiopharmaceutical path to the fractionator. The operator recognizes the correct valve through color identification (the brown valve flows to the fractionator) and by labeling the duct “Theo." Following an incident caused by a connection in another valve that caused a radiopharmaceutical to leak onto the equipment floor, the creation of this label became routine. Syringes used to circulate the radiopharmaceutical through the cassette are then connected. The operator describes what he evaluates in this step.

“I check for any obstructions in the plunger action of the syringe. Because they circulate the reagents, they cannot stop in the middle of the process, which has happened before. Then, while it is still inside the package, I pull and push the plunger; if it moves freely or does not lock, I install it; if not, I replace it with another syringe”. (Chagas synthesis operator).

Subsequently, to move the solution in the cassette, he checks whether the metal clips will fit over the plungers on the 30 ml syringes. A "click" sound that these clamps make when they are coupled to the syringes verifies this in addition to the visual inspection of the connection.

### 3.3 Eluent connection

The connection of the eluent (reagent responsible for transporting the radioisotope to the reactor flask, where the chemical reactions for the production of the radiopharmaceutical occur) was another stage of the cassette assembly that revealed a specific action to verify the accuracy of the assembly of the synthesis operator:

“You must be able to see the metal tip inside the eluent in order to determine whether it was spiked. […] To see the metal needle within the vial, you need to take a step back and make sure your viewing angle is straight. I am tall, therefore if you look down because of this, you have to stoop down and try to get your eyes 'straight' to the bottle before you can see the needle's tip inside.” (the Chagas synthesis operator).

### 3.4 Alignment and connection of additional reagents

In addition to the eluent, connections with other reagents must be carefully considered. The operator states that for all solutions to be sucked out, the needle should be placed in the lower third of the reagent bottle. He described an action that he developed from his experience when asked how he ensures that the bottle is in an ideal position:

“Just below the lower part of the ruler should be the lower part of the bottle cap. The ruler was originally intended to hold the cassette, but I modified it to also be used to place the flasks. I looked at what went wrong during quality control (to get to that position) and verified the installation of the bottle. For instance, I would return to the cassette assembly and check to see where the buffer solution had been if the radiopharmaceutical had become too acidic. With that insight, I understood that this was the perfect setting.” (the Chagas synthesis operator).

One of the flasks had a different fitting form, and the operator had to turn the flask rather than simply press it onto the needle during production room observations. He clarified:

“There is a greater chance that this flask will leak. It has previously occurred. Then, a radiopharmaceutical manufacturing unit informed us that if we rotated this vial while inserting it into the needle, it would not leak. It creates 'grooves' as if they were rubber folds when we turn it back and forth and descend. The reagent won't leak after that”. (the Chagas synthesis operator).

### 3.5 Absence of double-checking and visual confirmation of related components

The fractioning operator should be in contact when the cassette is fully assembled to perform a double-check. However, after realizing that the second check had not taken place, the operator remained in front of the machine for a short period. He clarified:

“I retrace my initial steps; everything is visual because everything is already in place. I begin with the connections. If I place the filter, if the FDG connection is connected to the path for transmitting it to fractionator, if all of the reagents are connected, if the eluent is attached, etc. It's easy to forget that the eluent is in a tiny vial of this size, right?” (Chagas synthesis operator).

He described this conference as a “stop" during the self-confrontation interview after identifying it during field observations. The synthesis operator double-checked the reported strategies and every step of the assembly process, mentally going through what he had done and ensuring that the cassette components were present.

He responded that setting up the fractionator “requires a lot of attention" and that interrupting his colleague could cause mistakes during the fractioning stage when questioned why he did not call the fractioning operator to check its assembly.

To better understand the lack of double-checking in the cassette assembly, the fractionation operator was also questioned (using cross-self-confrontation, with a video of the synthesis operator creating the cassette). He clarifies:

“Because the reagent's cap color matches the color of the position it takes up in the module, it is very difficult to make a mistake when using it. On the equipment, there seems to be a template with the colors and names of the components that need to be installed. For the operator to alter the colors, they would have to be fairly odd.” (the Lutz fractionation operator).

The fractionation operator explained why it is not necessary to check the cassette assembly and points out that it is simpler to do so because the equipment is designed with the materials to be connected, and the reagent flasks have different colors; that is, the vial cap color indicates where it should be plugged into the cassette. He contrasts his work with that of the fractionator to support his assessment of this facility:

“Since they are numbers and require attention, there must be double checking in the fractionator. Unlike the synthesis, the recipe does not have a test. Therefore, it's possible that radioactive activity presented incorrectly to customers. There is a higher likelihood of making mistakes because every day is a distinct dose fractionation recipe. The materials employed in synthesis are unaltered”. (Lutz fractionation operator).

This statement demonstrates his belief that the cassette assembly is “invariable" because the materials are the same. According to Lutz, the equipment features self-tests that obviate potential assembly mistakes (such as disconnected tubes).

Group discussions with substitute operators were organized to delve deeper into the topics already discussed. The production coordinator was also invited to participate in the discussions because he had knowledge of both synthesis and fractionation and could provide inputs to the discussions. The synthesis operator performed **self-checking** when no double-checking was performed. The operators were then informed of these findings and confronted with the existing in-place checks and their shortcomings, including double-checking, system checking, and self-checking.

### 3.6 Group discussions regarding conference procedures

Table 2 summarizes different evaluation processes based of the reports and discussions at different conferences, as reviewed by the operators.

**Table 1:** Data collection stages and respective participants, RPU, 2020–2021.

Stage	Goal	Participants
Initial virtual interviews	Understand how the RPU works and know the worker profiles	- Unit manager - Production coordinator
Open observations and self-confrontation interviews (Phase 1)	Analyze the activity of all operators in the synthesis and fractionation of radiopharmaceuticals and understand the relationship with the double conference	- Official synthesis operator - Official fractionation operator
Systematized observations and interviews in self-confrontation (Phase 2)	Deepen the analysis of the synthesis operator's activity and relate it to the need for double-checking	- Official synthesis operator
Self-confrontation interviews (Phase 3)	Debate and identify successful actions in radiopharmaceutical production	- Production coordinator - Official synthesis and fractionation operators - Substitute operators

Source: prepared by the authors.

**Table 2:** Comparison between possible evaluations by self-checking, double-checking and system checking, RPU, 2020–2021.

Assessed aspect	Self-checking	Double-checking	System checking
Tightening connections	The synthesis operator uses touch to feel the end of the rotation and identify the limit of force that must be applied.	Other operators would not be able to check, as the cassette is already connected to the equipment (if this were done, they would have to disconnect the cassette – which would take more time).	Through pressurization/depressurization, the system checks for possible leak points in the cassette.
Assessment of “knot”	The synthesis operator makes the “knot” in the rigid portion of the cassette and visually checks it immediately afterward.	Other operators carry out visual inspection (check if the knot was made).	The system does not identify the presence of the knot but would indicate an obstruction in the circuit if the knot was made in the flexible part.
Gas filter fitting	The synthesis operator performs a visual inspection (checks if the filter is installed) when stopping for verification (self-checking).	Other operators perform visual inspection (checks if the filter is installed).	The system does not indicate the presence of the filter. However, it indicates the location of the leak (in case of disconnection).
Fitting with the vial of radiometals	The synthesis operator performs visual inspection to identify the connection of this vial.	Other operators also perform visual inspection.	If this vial is not connected or is poorly connected, the system indicates a leak at the site.
Connection to the outlet for the fractionator	The synthesis operator performs the visual inspection (checks whether the track has been connected correctly – observes the label and color of the track).	Other operators perform visual inspection (checks whether the track has been connected correctly – observes the label).	The system cannot identify whether this path is connected.
Plunger locking of 30 ml syringes	The synthesis operator checks manually: the operator moves the syringe plunger while it is still in the packaging. When connected to the system, the operator evaluates whether the coupler will be able to move the plunger.	Other operators do not perform this check, as they believe that the equipment pressurizes enough to unlock the plunger.	The system is unable to verify that the plunger is locked.
30 ml syringe coupling	The synthesis operator hears the “click” of the clip that attaches to the syringe. The operator also visually inspects whether the clip is open.	Other operators perform visual inspection (they observe whether the clip is open) and auditory inspection (they also hear the “click”).	The system is unable to perform this check.
Coupling of the NaOH syringe	The synthesis operator swings the plunger and feels if it hits the sides of the coupler; he also bends down and observes whether the plunger is inside the coupler cavity.	Other operators do not check this step.	The system cannot perform this check.
Connecting the vials to the needles	The synthesis operator aligns the bottle cap with the bottom of the acrylic ruler (see item 3.4).	Other operators perform visual inspection (assess whether the needle is inside the vial).	The system is unable to perform this check.
Eluent connection	The synthesis operator bends down to view the needle inside the vial (see item 3.3).	Some operators report that they only do visual inspection (others press the vial toward the needle, to ensure that it has been punctured).	The system is unable to perform this check.
Formation of “grooves” in the rubber of the citrate vial	The synthesis operator secures the grooves by rotating the vial when connecting it to the needle.	The vial is already connected; thus, the operators are unable to perform this check.	The system is unable to perform this check.
Filling the gas waste bag	Based on the experience of the synthesis operator, he states that the gaseous bag supports up to two syntheses.	Other operators carry out visual inspection, evaluating whether it is fully inflated.	The system is unable to perform this check.
Reactor flask positioning	The synthesis operator performs a visual inspection to check whether the reactor flask is fitted to the module and whether the needle touches the bottom of the flask.	Other operators state that they do the same in the double-checking.	The system is unable to perform this check.

Source: Prepared by the authors.

This table is significant for discussing the constraints of various conference formats. It was also conceivable for the employees to determine which cassette assembly steps would be crucial, considering the amount of detail that the operators would need to pay attention to (and the fact that the automatic control of the system would not function as a backup) and the pattern of production issues. According to Freire (production coordinator), the connections for the eluent, reagents, and needles and that for the radiopharmaceutical and fractionator are crucial points.

Official operators evaluated the following crucial points regarding the work procedure from the examination of Table 1:

“The most crucial step is self-checking because it examines more of the cassette's assembly in depth. Next is double-checking, and last is system-checking.” (Lutz, fractioning operator).

“After the display of this image, a lot of things began to make sense, such as the problem with the eluent connection, which the system does not guarantee (if it was done).” (Chagas synthesis operator).

Contrary to his statement in the first interview, Lutz realized that the system has limitations in that the synthesis operator gets around by closely monitoring the processes occurring one after another to produce the radiopharmaceutical.

The unit experienced nonconformities as a result of its inability to double-check the suggested format. The operators determined that double-checking should take place at specific times, such as on audit days, when there may be cognitive interference in the self-checking process. This was done considering the perceived importance of audits, as demonstrated by the discussion of the activity analysis results. Currently, auditors interrupt operators’ responses to their inquiries. In particular, throughout the course of one and a half years, the only case of noncompliance resulting from a breakdown in the synthesis production process occurred when the eluent was not connected. The incident occurred in the morning of an audit in the production room. This suggests that auditors may have influenced the techniques created by the official synthesis operator and discussed in this paper. Operators claim that double-checking should be performed every time they return to the production area after being away for a certain period, such as after a vacation. In addition, double-checking would also be necessary if the operator is doubtful about how the cassette has been assembled in general

“When we returned from vacation, it would be interesting to continue the double check, at least until we established a production routine.” (Chagas, the official synthesis operator).

“When the operator is unsure about the assembly, a second inspection is also necessary.” (Freire, production coordinator).

Another finding from the discussions in the report was the operators' unawareness of Chagas’ self-checking procedure and the technique he uses to ensure that the vials are placed exactly where they should be (by aligning the vials' lids slightly below the acrylic ruler).

I was unaware of this Chagas technique. It seemed more useful to me … I'll adopt it as well. (Cruz, fractionation-substitute operator)

Chagas, does placing the vials in that position actually work? (Freire, production coordinator)

## 4 Discussion

The findings of this study indicate that conformity to norms should be viewed as a dynamic, multifaceted process with several levels of organization [26]. The operators did not entirely trust the automatization of the machine, contrary to what was stated in the RPU report. Subtle concerns begin with the cassette assembly, a task full of tacit knowledge [37] that is hard to express and frequently summed up in expressions that refer to the feeling or sensation of the body while engaging in an activity that makes this certainty possible: “it is in touch.” For everyone to agree to new internal rules during unforeseen circumstances (circuit leakage, radiopharmaceutical leakage on the equipment floor, or solution leakage), the norm had to be complemented and ingrained into the daily routine of a group of workers [35]: this included implementing the “knot,” making “grooves" in the citrate vials, and identifying the vial responsible for transporting radiopharmaceuticals to the fractionator, among other measures.

This study discussed criticisms of the ultrasafe model. In these systems, deviations are addressed systematically through audits, as exemplified in this study. However, despite being effective in identifying deviations, these audits may have blind spots in the investigative process as they do not encompass an understanding of the gap between the work-*as*-done and the work-*as*-imagined [6,9,38]. If deviation has become the new norm (and is informal), its predecessor must be discussed and transformed by all those involved in the real work. In this scenario, strengthened communication is the next step, as relevant information must circulate within the institution to ensure that operational actions align with the current knowledge of all workers involved [10,39,40]. Unlike the norms agreed upon by the collective in the past, self-checking was previously known only to the author. This serves as a warning sign for the need for clear communication to maintain a proactive collective.

Compliance with the rules is predicated on the idea that the operators' initiatives should be supported, as these are complementary and not antagonistic issues, as indicated in the theoretical questioning that guided this study. Operators will take ownership of the rules and display the compliance behavior sought by the business when they effectively participate in the design of new rules and the refinement of current rules. This interaction leads to the development of norms that are more sensible, adhere to a given reality, and are more effective [4].

In this case study, the process of developing and transforming a rule was conducted on four levels.

1) At the internal and external control system levels

The original team of operators at the investigated unit, majority of whom only temporarily replaced the current/official operators, performed double-checking when radiopharmaceuticals were first produced. Rule termination was a viable option. If there had been a second conference, would the cycle of effectiveness have ended? In addition to time constraints, noncompliance was identified by a team of internal employees who, in principle, would be more familiar with the specifics of the manufacturing process than an external audit team. This finding contradicts that of Bourrier [11], who demonstrated that internal audit teams typically exhibit greater adaptability and initiative when modifying the norm. In this study, the audit team judged conformity to norms without investigating the cause of variance.

2) At the collective level of work

Labor collectives are structured by a formal organization; however, they are also established to handle difficulties arising in the everyday manufacture of radiopharmaceuticals. The lack of a double conference reflects an informal agreement between the operators of dosage synthesis and fractionation. No external verification (double or alloconference) of the cassette assembly was performed based on the rationale that “there was no variability" in the materials employed. By contrast, typing in the division of doses was considered a more error-prone phase because the numbers changes daily; thus, it requires more attention and fewer distractions. Is it a sign of collective frailty? There was no record of significant accidents in the unit, which could imply a high level of operator and collective competency in dealing with various real work. When work collectives are in poor conditions, they detect fewer incidents, and communication is ineffective, which can harm the entire safety system and the health of its members [4,6]. Work collectives recognize and recover from anomalous situations and suggest more effective guidelines than those outlined in standard operating procedures. Control systems are constantly enhanced (or should be) through these collectives, which frequently create redundancies (double and/or self-conference) and integrate knowledge, experience, and cooperation.

3) In the face of unusual circumstances

Field observations revealed that double-checking would be impractical at times owing to the risk of disrupting the entire production process. For example, the delay in preparing the fractionation module could be attributed to the inadvertent shattering of the microbial growth plate, which disseminated the gelatinous substrate throughout the equipment. This resulted in a synchronization problem between the synthesis and fractionation modules (before the radiopharmaceutical is sent from the synthesis module to the fractionation module, it must be prepared with the dilution solution, and all vials must be positioned and labeled). The corresponding operator prepared the synthesis module after cleaning the fractionator to minimize the impact on the deadlines. If double-checking was performed on that day, production would have been further delayed, with direct consequences for the half-life of the radioisotope used and the amount of radiopharmaceuticals produced.

4) Within the context of the activity

Work activity corresponds to the complex mobilization of workers' capacities and the use of available means to attain their goals while considering real-world work settings [41]. Situations are prompt (new) behaviors [6,9]. Owing to the absence of double-checking, an activity known as self-checking was established separately (a scenario generated a new doing). Given the informal agreement on the lack of double-checking and the importance of verifying the assembly, a process of detailed re-inspection takes place, beginning with the assembly and mobilization of the synthesis operator's evaluation capacity in relation to the materials (testing the syringe plunger and tightening connections) and the process as a whole (ideal positioning of the reagent vial to stabilize the pH of the radiopharmaceutical).

**Fig. 4:**
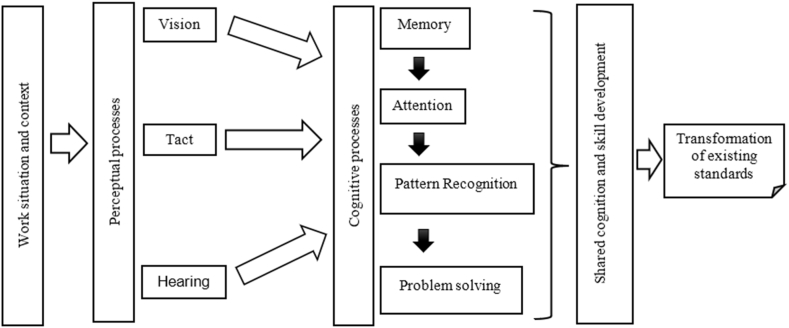
Perception and cognitive analysis in the conference processes for the radiopharmaceutical cassette assembly, RPU, 2020–2021 (Source: Prepared by the authors).

As shown in Fig. 4, emphasizing the role of the synthesis operator in transforming existing norms is crucial, as elucidated by the cognitive analysis of their activities. This operator perceives various stimuli from the work environment (observes the movement of the cassette parts, hears sounds, and feels the friction of the cassette components during assembly). Such information is processed and interpreted by cognitive processes (attention to details, memory of previous events, and problem solving) that provide indicators for action. Although illustrated separately, the perceptual and cognitive processes interact on a continuum [42]. This analysis clarified how they carried out their work by evoking knowledge and competencies, sometimes already shared by all and sometimes created for their actions.

Within the collective context, this shared cognition enhances the group's ability to act [43] as it enables the structuring of action through interaction among workers in each work context. Accordingly, the analysis of the existing conferences (double-checking, self-checking, and system checking) further enhanced the discussions on the application of norms to workplace conditions. Lutz (the fractioning operator) believed that the system would adequately ensure production and would not require an operator to check it, which permeated the initial interview. Such a claim indicated a certain illusion of control, according to which the system would have control over the course of events at work [4], allowing deviation from the established norm. However, the discussion of the activity analysis report allowed us to reflect on the necessity of double-checking and the significance of the synthesis operator's conference (self-checking). By identifying the circumstances in which double-checking might become part of reality and fit the demands of the job and operators, the debate enabled an adaptation of the norm [41].

Thus, the discussion on the divergence between norms and activities reveals a potential route for convergence. The deviations reflected by nonconformities at the control system level (via internal and/or external audits) represent the first indication that the gap between what is prescribed and what is possible may widen. Workers attempted to balance their demands with the activities, dangers, and profits involved on a collective basis. As demonstrated in this study, the double-checking activity must be tailored for certain situations because preventing the operator from performing his primary task to perform this evaluation could negatively impact productivity and safety (contradictory to the activity itself). This challenge increased the ingenuity and initiative of the synthesis operators as they modified the current checking to be completed by the operator who created the montage. Finally, this new verification and analysis process was formalized by discussing the different conference types and their limitations. However, even if it is followed at this time, this collective will need to be continuously evaluated in light of potential changes in verification, production, and work processes.

According to the findings, to facilitate the adherence of standards to the activity, ergonomic recommendations are centered on four primary actions:

The most experienced operators in the unit (in this example, the official synthesis operator Chagas and production coordinator Freire) wrote a script for groups of two and for self-checking. This script should not limit the experience and expertise of operators in spotting issues; rather, it should serve as a starting point for checking procedures during the radiopharmaceutical manufacturing process. Studying the crucial cassette assembly steps will enable the development of scripts with logical rules.

● Self-checking will be codified as a standard procedure, and double-checking should occur at times deemed crucial by the operators, such as audits, upon returning from vacation or time off from work, and whenever the synthesis operator feels uneasy about the assembly. The checking procedure must be constantly redundant to enhance the worker collective control system.
● To enable for conversation with other operators and to facilitate the exchange of experience and knowledge with regard to novel workplace conditions, a record book for events in the production room will be made. The group continuously learns; consequently, the group helps shape norms as they develop.

Regular meetings will also be organized (every 15 days or as needed) to discuss recorded incidents and improve group communication through discussions of rare circumstances.

However, as discussed in this study, their application necessitates the ongoing monitoring of potential adaptations. Notably, these recommendations should not be translated into fool-proof standards because they would not be able to account for every scenario when performing the activity [2,9,10,44].

According to the clinical perspective of the activity, the outcomes allowed for dialogic action at work [34].

It entails starting a discussion between experts [ …], centered around a video of the action, in order to recover disagreements regarding the activity inside the group and among "connoisseurs." These "disputes" are intended to restore the ability of those engaged in trade to take action, thus strengthening the collective guarantee of individual activities [34].

Regarding adherence to norms, it also became clear that frequent renormalization is necessary in real work contexts, including a combination of appropriating the antecedent norm for action and its revision (adaptation) [27].

It is important to note that this study assumed conditions in which the production team was complete. The strategies to be developed in the event of a reduction in the official team were not investigated; thus, its impact on radiopharmaceutical production and the health of workers are also unknown. One limitation of this study is that field observations were performed with the official team of operators, as their substitutes had risk factors for on-site work during the COVID-19 pandemic, preventing analysis with different worker pairings. In addition, this study did not analyze possible communication barriers among operators, leading to the informality of a more context-adherent strategy.

## 5 Conclusion

This study examined the adherence to norms of activities using noncompliance as a starting point. A self-checking method ensures product delivery, even though there is no double-checking during the radiopharmaceutical cassette assembly. According to the records of the unit, a single instance of noncompliance stemming from a production failure caused by a cassette assembly problem occurred on an audit day when the auditors might have interrupted the operator's checking strategies.

Activity analysis allowed us to determine that the workplace is standardized in various ways. Managers claim that one should not believe in the automation of machines. However, the investigation revealed that the official synthesis workers constantly paid attention to how the equipment was operating to take appropriate action to prevent and/or correct failures. In addition to highlighting the operator's crucial role in providing the product, this study emphasizes the disconnect between managers' ideas and the workplace realities. This disconnect resulted in repeated noncompliance and recommendations of actions (operator training) that had no impact.

Finally, a discussion of the rules and protocols within the context of work collectives is necessary to search for conformity in such a standardized environment. Understanding the task and the gap between the prescribed and real work is necessary to widen the previously established safety margins. The work groups' established communication, experience, and control systems may enable daily learning to handle unforeseen circumstances. Consequently, it is possible to constantly address system flaws and improve group rules.

The results of this study contribute to the discussion on workplace safety in contexts characterized by standardization and advance the discussion on changing perspectives regarding rule compliance (emphasizing the importance of analyzing work activity and the collective of workers). In work scenarios in which the details and time constraints of an activity influence the worker's actions, discussing ways to develop the work and its respective rules is essential. In making a norm more adherent by analyzing the context at four levels (external and internal control, the collective of workers, work situations, and work-*as*-done), this study highlighted a possible methodological path for transforming work. In future research, we will highlight the importance of addressing the benefits of ergonomic work analysis at other stages of radiopharmaceutical production, such as dose fractionation and quality control.

## Research data for this article

Due to the sensitive nature of the questions asked in this study, survey respondents were assured raw data would remain confidential and would not be shared.

## Data not available

The data that has been used is confidential.

## Ethics declarations

This study was reviewed and approved by Research Ethics Committee of the René Rachou Institute – Fiocruz Minas, with the approval number: CAAE 30808020.6.0000.5091.

All participants provided informed consent to participate in the study.

All participants provided informed consent for the publication of their anonymised case details and images.

## CRediT authorship contribution statement

**Gerferson André Silva Costa:** Writing – review & editing, Writing – original draft, Visualization, Validation, Project administration, Methodology, Investigation, Formal analysis, Data curation, Conceptualization. **Julie Micheline Amaral Silva:** Writing – review & editing, Validation, Methodology. **Raquel Guimarães Soares:** Writing – review & editing, Supervision, Methodology. **Celina Maria Modena:** Writing – review & editing, Supervision, Methodology. **Fabiana Goulart de Oliveira:** Writing – review & editing, Supervision, Methodology. **Francisco de Paula Antunes Lima:** Writing – review & editing, Supervision, Methodology, Formal analysis.

## Declaration of competing interest

The authors declare that they have no known competing financial interests or personal relationships that could have appeared to influence the work reported in this paper.

## Footnotes

1 Companies create golden rules in an attempt to differentiate between procedures in the sea of regulations that submerges businesses. Dekker adopts a more radical stance and proposes “The safety anarchist” [1].

2 “The participation of operators in improving the rules and procedures aims not only to favor their applicability, but also helps to develop their appropriation by the operators. This increases their intrinsic motivation to apply them, with the support and help of the team. As a result, when the participatory plan is well done, it contributes to reinforcing the rigor of the application.” [4].

3 This study was approved by the Research Ethics Committee of the René Rachou Institute – Fiocruz Minas, under number CAAE 30808020.6.0000.5091, on 06/12/2020, in compliance with the fundamental aspects of Resolution 466/2012 of the National Health Council. In order to preserve the confidentiality of the data, the workers interviewed in this research were given surname of scientists as pseudonyms.

4 The cassette is a set of tubes, reagent bottles, valves, syringes and filters that is coupled to the synthesis module for the production of the radiopharmaceutical. Each cassette has a bar code that makes it possible to track the batch produced. After connecting it to the module, the synthesis of the radiopharmaceutical by the cassette is controlled and monitored by software that automates the process and reduces the worker's exposure to radiation [36].
